# Human Pulmonary Dirofilariasis Due to *Dirofilaria immitis*: The First Italian Case Confirmed by Polymerase Chain Reaction Analysis, with a Systematic Literature Review

**DOI:** 10.3390/life12101584

**Published:** 2022-10-12

**Authors:** Andrea Palicelli, Claudia Veggiani, Francesco Rivasi, Andrea Gustinelli, Renzo Boldorini

**Affiliations:** 1 Pathology Unit, Azienda USL–IRCCS di Reggio Emilia, 42123 Reggio Emilia, Italy; 2 Pathology Unit, Azienda Ospedaliero-Universitaria Maggiore Della Carità, 28100 Novara, Italy; 3 Pathology Unit, University of Modena and Reggio Emilia, 41124 Modena, Italy; 4 Department of Veterinary Medical Sciences, Università di Bologna, 40064 Bologna, Italy; 5 Department of Health Sciences, University of Eastern Piedmont (UPO), 28100 Novara, Italy

**Keywords:** *Dirofilaria*, *immitis*, dirofilariasis, zoonosis, Italy, human, polymerase chain reaction, PCR, lungs, pulmonary

## Abstract

Dirofilariasis is a zoonosis caused by nematodes of the genus *Dirofilaria.* *Dirofilaria immitis* is cosmopolitan as regards its distribution in animals, being responsible for human pulmonary dirofilariasis in the New World. However, human infections by *Dirofilaria immitis* are exceptional in Europe, and the previously reported Italian cases of pulmonary dirofilariasis were due to *Dirofilaria repens*. We performed a systematic literature review of the Italian cases of human dirofilariasis due to *Dirofilaria* *immitis* according to the PRISMA guidelines. We also report the first autochthonous case of human pulmonary dirofilariasis due to *Dirofilaria* *immitis*, confirmed by polymerase chain reaction analysis. The patient was a 60-year-old man who lived in the Po river valley and had never traveled abroad; on histological examination, the 2-cm nodule found in his right upper lung was an infarct due to a parasitic thrombotic lesion. Only one other autochthonous (but conjunctival) case due to *Dirofilaria* *immitis* (molecularly confirmed) was previously found in the same geographic area. Climatic changes, the increasing movements of animal reservoirs and vectors, and new competent carriers have expanded the geographic distribution of the *Dirofilaria* species, increasing the risk of human infections. Our report demonstrates that at least some pulmonary Italian cases of human dirofilariasis are due to *Dirofilaria immitis*, as in the New World.

## 1. Introduction

Dirofilariasis is a zoonosis caused by nematodes of the genus *Dirofilaria* (Filarioidea: Onchocercidae) [1,2,3,4,5,6,7,8,9,10,11,12,13,14,15,16,17,18,19,20,21,22,23,24,25,26,27,28,29,30,31,32]. *Dirofilaria repens* (*D. repens*) and *Dirofilaria immitis* (*D. immitis*) are common mosquito (*Culicidae*)-transmitted parasites found in Canidae and felines, and the most frequently involved species in human dirofilariasis [1,5,6,10,11,12,13,14]. Climatic changes, the increasing movements of animal reservoirs and vectors, and new competent carriers (such as *Aedes Albopictus*) have expanded their geographic distribution, increasing the risk of human infections [16,17,18,19,20]. *D. repens* is smaller, has an undulated external cuticle, and seems to be present only in the Old World. Conversely, *D. immitis* is larger with a smooth cuticle, and is cosmopolitan as regards its distribution in animals, while human infections in Europe are exceptional [1,3,5,6,8,9,16,17,18,19,20]. We report the first Italian case of autochthonous human pulmonary dirofilariasis due to *D. immitis*, confirmed by polymerase chain reaction (PCR) analysis. We have also performed a systematic literature review of the Italian cases of human dirofilariasis due to *D. immitis*.

## 2. Materials and Methods

The PCR analysis was performed on a 10% buffered formalin-fixed, paraffin-embedded tissue sample (Pathology Unit archive material), as previously described [1,2]. Briefly, 4 μm-thick tissue sections were placed in 1.5 mL Eppendorf tubes after selection of the area of interest by an expert pathologist, thereby enriching the sample by increasing the percentage of parasitic material. The DNA was extracted using EDTA-SDS/proteinase K treatment (Celbio, Milano, Italy) followed by phenol–chloroform, and re-suspended in 50 μL of diethylpyrocarbonate-treated, autocleaved pyrogen and RNAse-free water (Eppendorf Milano, Italy); 10 μL of extracted DNA was added to the PCR mixtures. The human beta-actin gene was used as a positive control for DNA extraction to avoid false-negative results in the PCR analysis.

The sequence of the tested PCR primer sets was retrieved by the GenBank database (https://www.ncbi.nlm.nih.gov/genbank/; accessed on 10 October 2022). The primers included:

- *D. immitis*: 5′-ACGTATCTGAGCTGGCTCAC-3′ (forward, position: 13–32) and 5′-ATGATCATTCCGCTTACGCC-3′ (reverse, position: 390–371) (GenBank database, accession number M95173; https://www.ncbi.nlm.nih.gov/nuccore/M95173; accessed on 10 October 2022), specific to a 378 base-pair fragment of the cuticular surface antigen gene of *D. immitis*.
- *D. repens*: DIR 3 (5′-CCGGTAGACCATGGCATTAT-3′, forward, position: 54–73) and DIR 4 (5′-CGGTCTTGGACGTTTGGTTA-3′, reverse, position: 299–280) (GenBank database, accession number L15323; https://www.ncbi.nlm.nih.gov/nuccore/L15323; accessed on 10 October 2022), specific to a 246 base-pair fragment of IpS insert 11 of *D. repens*.

We searched and aligned the GenBank sequences to a query sequence that included *Dirofilaria repens*, *Dirofilaria immitis* and the abovementioned accession numbers using BLAST (Basic Local Alignment Search Tool) (https://blast.ncbi.nlm.nih.gov/Blast.cgi; accessed on 10 October 2022). The primers were synthesized by Roche Diagnostics, S.p.A., Monza, Italy; they have been commonly and successfully used in various previously reported studies to specifically identify these *Dirofilaria* species [1,2,20,21,22,23]. Negative and positive tissue controls (i.e., other previously diagnosed and PCR-confirmed *D. repens* and *D. immitis* cases) were also tested by PCR analysis for both the *Dirofilaria* species. Further information about the PCR analysis (including the details of the reaction mixture and thermal cycling phases/timing) are described in Table 1 and Table 2.

**Figure 1:**
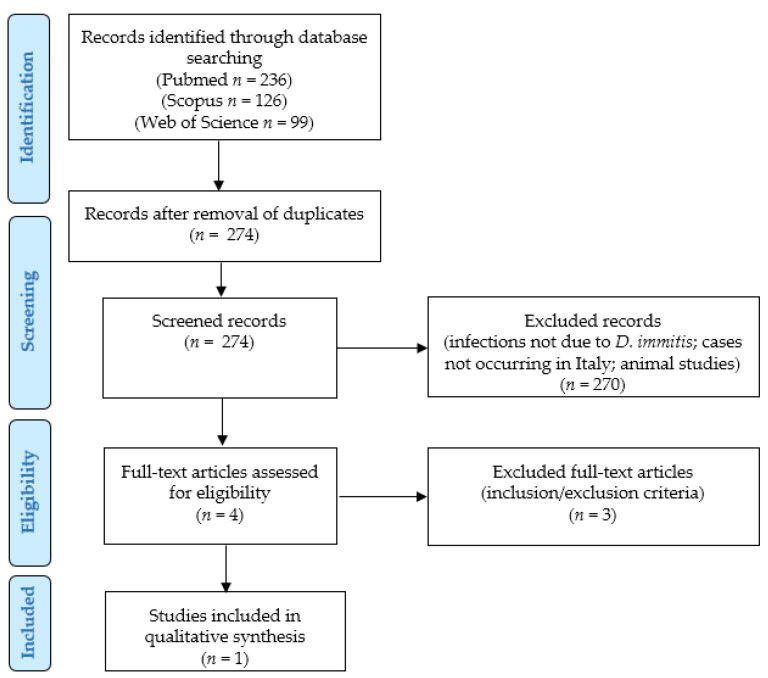
Systematic literature review: PRISMA flow chart.

The systematic literature review of our retrospective observational study was conducted according to the “Preferred Reporting Items for Systematic Reviews and Meta-Analyses” (PRISMA) guidelines (http://www.prisma-statement.org/; accessed on 10 October 2022) in order to identify cases of human infection by *D. immitis* in Italy (Figure 1).

We answered the following PICO questions:

- (P)opulation: human patients affected by infections due to *D. immitis* in Italy;
- (I)ntervention: any;
- (C)omparison: none;
- (O)utcomes: patients’ clinical outcomes (status at last follow-up, response to therapy, and survival and recurrence rates); detection rate of PCR analysis and histopathological exam.

We searched for (dirofilaria OR dirofilariasis OR dirofilariasi OR dirofilariosis OR “D. immitis” OR “D.immitis”) AND immitis AND (Italy OR Italian) in the PubMed (all fields; 236 results; https://pubmed.ncbi.nlm.nih.gov, accessed on 10 October 2022), Scopus (Title/Abstract/Keywords; 126 results; https://www.scopus.com/home.uri, accessed on 10 October 2022) and Web of Science (Topic/Title; 99 results; https://login.webofknowledge.com, accessed on 10 October 2022) databases. No limitations or additional filters were set. The bibliographic research ended on 10 October 2022. We applied the following:

- Eligibility/inclusion criteria: case series/reports of human dirofilariasis due to *D. immitis* in Italy.
- Exclusion criteria: infections not due to *D. immitis*; cases that did not occur in Italy; animal studies.

After excluding duplicates, two independent authors checked the titles and abstracts of all the retrieved results, applying the eligibility, inclusion, and exclusion criteria. They selected 4 relevant eligible papers, all of which were obtained in full-text format and screened for additional references and to verify the inclusion criteria. After reading the full text of each of the 4 papers, 3 of them were excluded for not meeting the inclusion criteria. Two other authors checked the extracted data, and 1 article was finally included in our study [3]. Data collection was study- and case-related. As the included article reported 1 case, we present a simple summary of this case; no particular statistical analyses could be performed.

## 3. Results

### 3.1. Case Report

A 60-year-old man, living in the Po river valley, presented at the Modena city hospital with persistent, low-grade fever. Cough, thoracic pain, and hemoptysis were absent. He had never traveled abroad. An X-ray exam revealed a peripheral nodule in the right upper pulmonary lobe. Computed tomography scans confirmed a well-delimited nodule (maximum size: 2 cm). A pulmonary tumor was suspected, and a right upper lobectomy was performed.

On gross examination, the nodule was white-yellow, showed increased consistency, and measured 2 cm × 1.5 cm × 1 cm.

**Figure 2:**
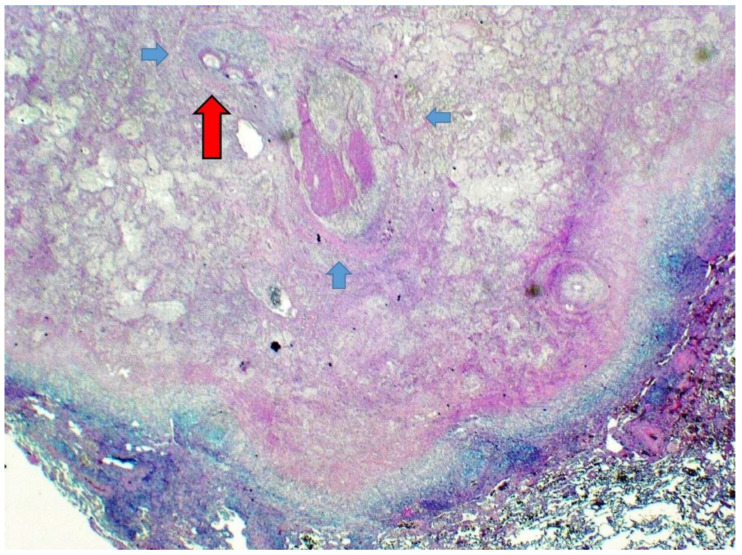
The pulmonary parenchyma revealed a nodular infarct surrounded by mild inflammation. A few sections of an adult nematode (long red arrow) were found in a structure with an undulated profile (probably a dilated blood vessel) (short blue arrows) (Masson’s Trichrome staining; 4×; previously unpublished, original photo).

**Figure 3:**
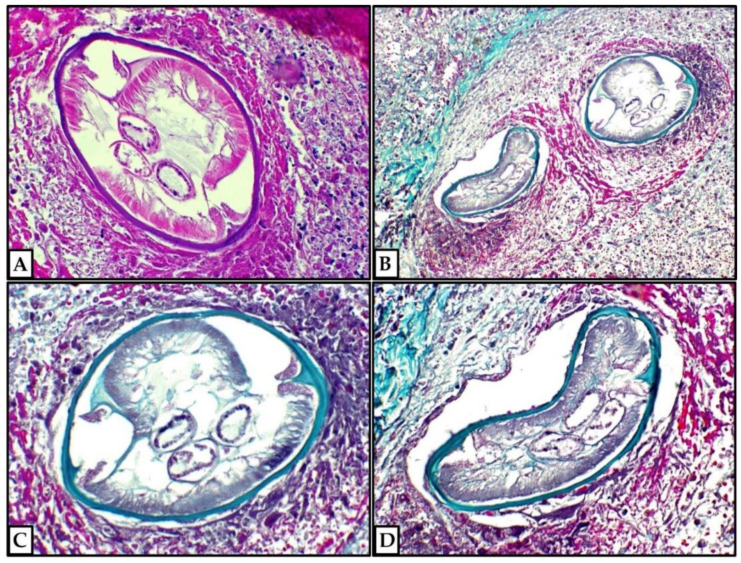
Morphologic details of *D. immitis*. The parasitic sections showed a smooth external cuticular surface. The internal organs (muscle tissue and intestine) were well preserved. (**A**) Hematoxylin and eosin, 20×; (**B**) Masson’s Trichrome, 10×; (**C**,**D**) Masson’s Trichrome, 20× (previously unpublished, original photos).

Histologic examination (Figure 2 and Figure 3) revealed a pulmonary infarct surrounded by slight fibrosis and mild inflammation.

The infarcted pulmonary architecture was still partially recognizable, revealing a parasitic thrombotic lesion in a pulmonary vessel (probably an artery). The few, well-preserved nematode sections were more visible after a few recuts and Masson’s Trichrome staining. Size and morphology, including the typical smooth profile of the external cuticle, qualified the nematode as a *D. immitis*. The PCR analysis confirmed our histological diagnosis, detecting the amplification of a 378 base-pair product specific to *D. immitis* DNA, while the PCR test for *D. repens* was negative; in all the performed tests, the positive and negative tissue controls resulted as expected. The patient recovered well after surgery, but we lost him at follow-up.

### 3.2. Systematic Literature Review Results

Only 1 case [3] was clearly autochthonous, as the patient did not travel abroad in the previous 8 years. It was a live male *D. immitis* (length: ~100 mm; thickness: 330 μm; smooth cuticle) extracted from the right eye (temporal bulbar subconjunctival space) of a 51-year-old Caucasian man, living in the city of Carpi (near Modena). The nematode caused foreign body sensation and conjunctival swelling. Other subcutaneous or pulmonary localizations, as well as microfilaremia, were excluded. The patient completely recovered. PCR analysis of the frozen worm parts (−80 °C) confirmed the diagnosis. Another conjunctival infection by *D. immitis* (an imported case in a Tunisian boy) did not undergo PCR analysis [4]. No convincing cases of pulmonary dirofilariasis due to *D. immitis* were previously reported in Italy.

## 4. Discussion

Most of the worldwide-reported human cases of dirofilariasis have been due to *D. repens*, which typically migrates through the tissues, resulting in subcutaneous, ocular/peri-ocular/conjunctival or deep soft tissue/visceral nodules [1,2,3,4,5,6,7,8,9,10,11,12,13,14,15,16,17,18,19,20,21,22,23,24,25,26,27,28,29,30,31,32].

Globally, human pulmonary dirofilariasis has rarely been described, typically due to *D. immitis*, which relatively prevails in human patients living in the New World and Japan. Conversely and exceptionally, this species caused human infections in Europe, but an extrapulmonary *D. repens*-like presentation is uncommon [1,2,3,4,5,6,7,8,9,10,11,12,13,14,15,16,17,18,19,20,21]. Mosquitoes release the filariae into the host, and the few survivors migrate to the pulmonary circulation, causing small (1–3 cm), well-delimited pulmonary infarcts with a granulomatous reaction. Frequently appearing as coin lesions on radiological exams (especially on computed tomography scans), these pulmonary nodules are easily confused with primary or metastatic pulmonary tumors (as in our case) [5,6,16,17,18].

However, the rule of the different behavior of *D. repens* and *D. immitis* is not universal, and important caveats are also linked to the epidemiologic distribution of the two species; subcutaneous cases due to *D. immitis* and pulmonary localizations of *D. repens* have rarely been described [1,3,5,16,17,18,19,20].

In Italy, over the course of several decades, the prevalence of *Dirofilaria* species has increased in endemic regions, as well as in areas where only sporadic cases had been previously reported; moreover, new animal and human cases have been described in formerly spared areas. In the endemic Po river valley, the prevalence of *D. repens* and *D. immitis* infections in Canidae reaches about 30% and >50%, respectively, while that of *D. immitis* in felines accounts for 7–27% [5,7,10,11,12,13,15].

Human infections due to *D. repens* seem to prevail even in European/Italian areas in which *D. immitis* is highly endemic in mosquitos and animal reservoirs. Indeed, the rare previously reported Italian cases of pulmonary dirofilariasis were due to *D. repens*, while our case represents the first one caused by *D. immitis*; moreover, considering the cases that occur in other sites of the body, this is only the second autochthonous Italian report of molecularly confirmed human dirofilariasis due to *D. immitis* [1,8,9,16,17,18].

Pulmonary, subcutaneous, or soft tissue nodules of dirofilariasis are usually incidentally found; after a clear histopathological diagnosis is established with or without the aid of molecular tests, most patients present no particular clinical complications at follow-up [1,3,16,17,18,19,20,21,22,23,24,25,26,27,28,29,30,31,32].

Our current knowledge about the prevalence rates and geographic distribution of the various *Dirofilaria* species, as well as their patterns of infection in human and animal hosts, could be affected by relevant diagnostic biases, concerning the clinical–pathological under-recognition of these parasitic lesions and the difficulties in correctly classifying the *Dirofilaria* species [1,3,16,17,18,19,20].

At least in some cases, worm sections are not easily detectable or classifiable upon histological examination, owing to their advanced necrotic stage or their unfavorable orientation for a proper evaluation of the external cuticle. Further recuts or Masson’s Trichrome staining may allow for an easier detection of the parasitic sections and a more contrasted evaluation of the different internal organs of these nematodes [1,3,5,8,9,16,17,18,19,20].

The identification of the correct *Dirofilaria* species might be difficult for general pathologists, who are usually not particularly confident with the histomorphological features of these parasites. Some authors [17] have critically reviewed the histological cases of human dirofilariasis attributed to *D. immitis* in the Old World, finding that at least some worms were incorrectly classified in the original reports, or information was too scant to properly identify the parasite species. Moreover, other patients with a diagnosis of infection by *D. immitis* were travelers/globetrotters, or lived outside Italy; therefore, it was impossible to establish if they had contracted the infection in Italy or abroad [17].

In addition, some cases were attributed to *D. immitis* by serological results without testing the serology of *D. repens* or lacking a histopathological confirmation of the parasitic nodule [17,18]. Recently, some authors concluded that the potential cross-reactivity of serological tests advocates for an integrative diagnosis with a morphological and molecular approach, given that the use of a sole serological test can be misleading or less specific/sensitive [15].

Molecular typization by PCR has been recently introduced and it is currently available only in selected laboratories; moreover, molecular analysis was not performed in older cases reported in the literature [1,3,5]. PCR is relatively cheap and may be performed on the archive samples of any Pathology Units, being particularly helpful when the parasitic sections are morphologically inevaluable [1]. The wider use of PCR may help in the proper definition of the epidemiologic distribution and behavior of the *Dirofilaria* species. Our report demonstrates that at least some pulmonary autochthonous Italian cases of human dirofilariasis can be due to *D. immitis*, as in the New World.

## Figures and Tables

**Table 1:** Polymerase chain reaction analysis: reaction mixture.

Reaction Mixture	Dirofilaria Species (Tissue Sample)	β-Actin
H_2_O	as required	as required
MgCl_2_	1.5 mM	3 mM
NH_4_ buffer	1×	1×
dNTPs	0.2 mM	0.2 mM
Primer (F)	0.5 pmol/μL	0.2 pmol/μL
Primer (R)	0.5 pmol/μL	0.2 pmol/μL
Taq DNA polymerase	0.075 U/μL	0.05 U/μL
DNA	10 μL	10 μL
Final volume	25 μL	25 μL

dNTPs: deoxynucleotide triphosphates; F: forward oligonucleotide; R: reverse oligonucleotide.

**Table 2:** Polymerase chain reaction analysis: thermal cycling phases and timing.

Polymerase Chain Reaction Phase	*Dirofilaria repens*	*Dirofilaria immitis*	β-Actin
First denaturation	94 °C for 5 min	94 °C for 5 min	94 °C for 5 min
Denaturation	94 °C for 30 s	94 °C for 15 s	94 °C for 30 s
Annealing	51 °C for 30 s	55 °C for 40 s	66 °C for 30 s
Extension	72 °C for 25 s	72 °C for 40 s	72 °C for 45 s
Number of cycles	48	40	35
Final extension	72 °C for 5 min	72 °C for 5 min	72 °C for 5 min
